# Quality of Visual Cue Affects Visual Reweighting in Quiet Standing

**DOI:** 10.1371/journal.pone.0150158

**Published:** 2016-03-03

**Authors:** Renato Moraes, Paulo Barbosa de Freitas, Milena Razuk, José Angelo Barela

**Affiliations:** 1 School of Physical Education and Sport of Ribeirao Preto, University of Sao Paulo, Ribeirao Preto, SP, Brazil; 2 Institute of Physical Activity and Sport Sciences, Cruzeiro do Sul University, Sao Paulo, SP, Brazil; 3 Laboratory of Movement Studies, Department of Physical Education, Sao Paulo State University, Rio Claro, SP, Brazil; Centre de Neuroscience Cognitive, FRANCE

## Abstract

Sensory reweighting is a characteristic of postural control functioning adopted to accommodate environmental changes. The use of mono or binocular cues induces visual reduction/increment of moving room influences on postural sway, suggesting a visual reweighting due to the quality of available sensory cues. Because in our previous study visual conditions were set before each trial, participants could adjust the weight of the different sensory systems in an anticipatory manner based upon the reduction in quality of the visual information. Nevertheless, in daily situations this adjustment is a dynamical process and occurs during ongoing movement. The purpose of this study was to examine the effect of visual transitions in the coupling between visual information and body sway in two different distances from the front wall of a moving room. Eleven young adults stood upright inside of a moving room in two distances (75 and 150 cm) wearing a liquid crystal lenses goggles, which allow individual lenses transition from opaque to transparent and vice-versa. Participants stood still during five minutes for each trial and the lenses status changed every one minute (no vision to binocular vision, no vision to monocular vision, binocular vision to monocular vision, and vice-versa). Results showed that farther distance and monocular vision reduced the effect of visual manipulation on postural sway. The effect of visual transition was condition dependent, with a stronger effect when transitions involved binocular vision than monocular vision. Based upon these results, we conclude that the increased distance from the front wall of the room reduced the effect of visual manipulation on postural sway and that sensory reweighting is stimulus quality dependent, with binocular vision producing a much stronger down/up-weighting than monocular vision.

## Introduction

Postural control functioning is characterized by flexible and adaptive accommodations to sensory changes. Indeed, studies have shown that the central nervous system modifies postural responses based upon continuing selection of sensory inputs that provide the most reliable information, named sensory reweighting [1,2,3]. Sensory reweighting is a non-linear process since that sensory integration is not simply a summation of inputs from different sensory systems [4,5,6]. Also, sensory reweighting is considered a dynamical process in which the postural control system identifies and selects the sensory inputs that provide the most useful information for achieving the goals of orientation and equilibrium [4,5].

Vision plays a fundamental role in the multisensory control of upright body position [7,8] and visual cues are dynamically combined with sensory cues from other sources [9], providing meaningful information to postural control system in order to produce appropriate postural responses. However, it has been shown that the visual contribution depends on whether visual cues are mono or binocular [10,11,12]. For instance, vision attenuates the effects of galvanic vestibular stimulation during quite standing tasks, but such effect is lower in the mono than in the binocular vision condition [10]. Specifically, visual cues were down weighted in monocular condition, indicating that information furnished by monocular cues is insufficient to guarantee a reliable reference frame for postural control and, thus, its weight is reduced. A possible explanation for this reduction is that occlusion of one eye alters depth cues and compromises visual field size, which may explain the reduced influence of vision for postural control under monocular condition.

The effect of monocular vision on postural control was also assessed in adults [11] and children [12] using the moving room paradigm, in which participants stood as quiet as possible in a room that was continuously moved back and forth while the floor remained stationary. This kind of visual manipulation changes the optical flow and induces postural adjustments such that individuals sway in the same direction and frequency of the visual stimulus provided by the moving room [13]. A recent study showed that body sway caused by the movement of the room was attenuated under monocular vision as compared to binocular vision [11]. This result is in agreement with the results obtained by Jessop and McFadyen [10], suggesting that the coupling between room movement and body sway, which is usually strong and stable with binocular vision [13], has its strength reduced in the monocular condition.

We used the monocular and binocular vision as a paradigm to study the dynamic properties of the visual reweighting process. The control of the amount of visual information allows us to identify how fast the reweighting process for visual information is. In our previous study [11], the visual condition was set a priori and participants were able to determine the approximate contribution of all sensory channels beforehand. However, this adjustment of the contribution of each sensory channel cues occurs dynamically while performing the ongoing postural task [3,4,5]. It is also known that previous knowledge of the characteristics of the room movement is sufficient to reduce the coupling between visual information and body sway [1,13,14], suggesting that cognitive processes can modulate the influence of optic flow on body sway [13,15,16]. Therefore, the unexpected change in visual condition during a postural task performed within the moving room allows one to investigate the dynamic process of visual weighting information. Thus, we investigated the dynamical adaptation of the visual information weight during the transition between binocular and monocular vision and vice-versa during continuous change in the optical flow caused by the room movement. To expand on the understanding of this dynamical adaptation, no-vision condition was also included in order to examine how the first exposure to room movement affects body sway.

Another aspect that affects the visual influence on postural sway is the distance of the individual to a target [7,17] or to the front wall of the moving room [11,13,18]. The shortened distance from the target for gaze fixation results in reduced body sway, due to a better resolution to detect relative movement of the head. For the moving room, the increased distance from the front wall decreases the coupling between room movement and body sway. In addition, the combination of monocular vision and increased distance from the front wall reduced this coupling [11]. Despite this reduction in coupling, the temporal relationship between room movement and body sway is constant for different distances [13] and for different visual conditions [11]. Therefore, the distance between the individual and the visual target placed in the front wall can also play a role in the dynamic adaptation due to change in the visual condition. Since increased distance attenuates the coupling between visual information and body sway, it could be hypothesized that adaptation would be lower and slower for the farther distance.

The purpose of this study, therefore, was to examine the effect of visual transitions in the room movement and body sway coupling in two different distances from the front wall of a moving room. For that purpose, it was employed an experimental procedure which involved participants wearing goggles with crystal lenses that allowed changes in lenses status from opaque to transparent and vice-versa in less than 5 ms while they stood within the moving room. When the lenses are opaque, it eliminates information about form and movement, but it maintains a level of unspecific light, which avoids adaptation due to light after lenses aperture. The lenses can also be controlled independently, which allows the experimenter to control monocular, binocular and no-vision conditions.

## Material and Methods

### Participants

Eleven healthy young adults (18 to 30 years-old) participated in this study (6 F e 5 M; 22.0±3.2 years-old; 1.69±0.69 m; 72.2±13.7 kg). Participants did not report any neurological, muscular, or orthopedic disorders that could affect their performance in this study and had normal or corrected-to-normal vision. Participants provided their written informed consent to participate in this study. The experimental protocol was approved by the Research Ethics Committee of the Cruzeiro do Sul University (Approval number: 162/2009).

### Procedures

Participants were instructed to stand as quiet as possible within a moving room facing the front wall. This moving room consists of three walls (2.1 m x 2.1 m x 2.1 m) and a ceiling mounted on wheels that can oscillate in the anterior-posterior (AP) direction, controlled by a servomotor mechanism. The ceiling was coated white and the walls were coated with a pattern of alternating white (42 cm wide) and black (22 cm) stripes, enhancing contrast information. The servomotor mechanism consisted of a controller (Model APEX 6151, Compumotor, CA, USA), a controlled stepper motor (Compumotor, CA, USA), a software program that controlled the motor outputs (Motion Architect for Windows, Compumotor, CA, USA), and an electrical cylinder (Compumotor, CA, USA) that connected the servomotor mechanism to the moving room. Note that only the walls and ceiling moved back and forth, while the ground remained stationary. A fluorescent lamp (20 W) was placed on the room’s ceiling to keep the room illuminated by constant light.

The room oscillated at a frequency of 0.2 Hz, which is approximately the natural human body sway in a quiet standing position [19], with peak velocity of 0.75 cm/s and peak-to-peak amplitude of 1.2 cm. An infrared emitting diode (IRED) was placed on the back of the participant between scapulae at approximately 8^th^ thoracic vertebrae. Other IRED was placed on the front wall of the moving room. An OPTOTRAK™ camera (Northern Digital, Waterloo, Canada) was positioned behind participants and monitored IREDs displacement at a sampling frequency of 100 Hz.

Participants stood in the middle of the moving room relative to the lateral walls at two distances from the front wall, 75 and 150 cm, barefooted with feet apart at shoulder distance, and with upper limbs relaxed along the body. During each trial, participants were asked to fixate at a target (black circle with 5 cm of diameter) placed at eye level in the front wall of the moving room. During the experimental procedures, participants wore a pair of liquid crystal display (LCD) goggles (Translucent Technologies Inc., Toronto, Canada) that allows lens status transition from opaque to transparent and vice-versa in approximately 5 ms, with right and left lens controlled independently. Lens status was controlled by a routine written in LabVIEW (version 2010, National Instruments, Inc). Participants, when necessary, wore their own glasses together with the LCD goggles.

**Fig 1:**
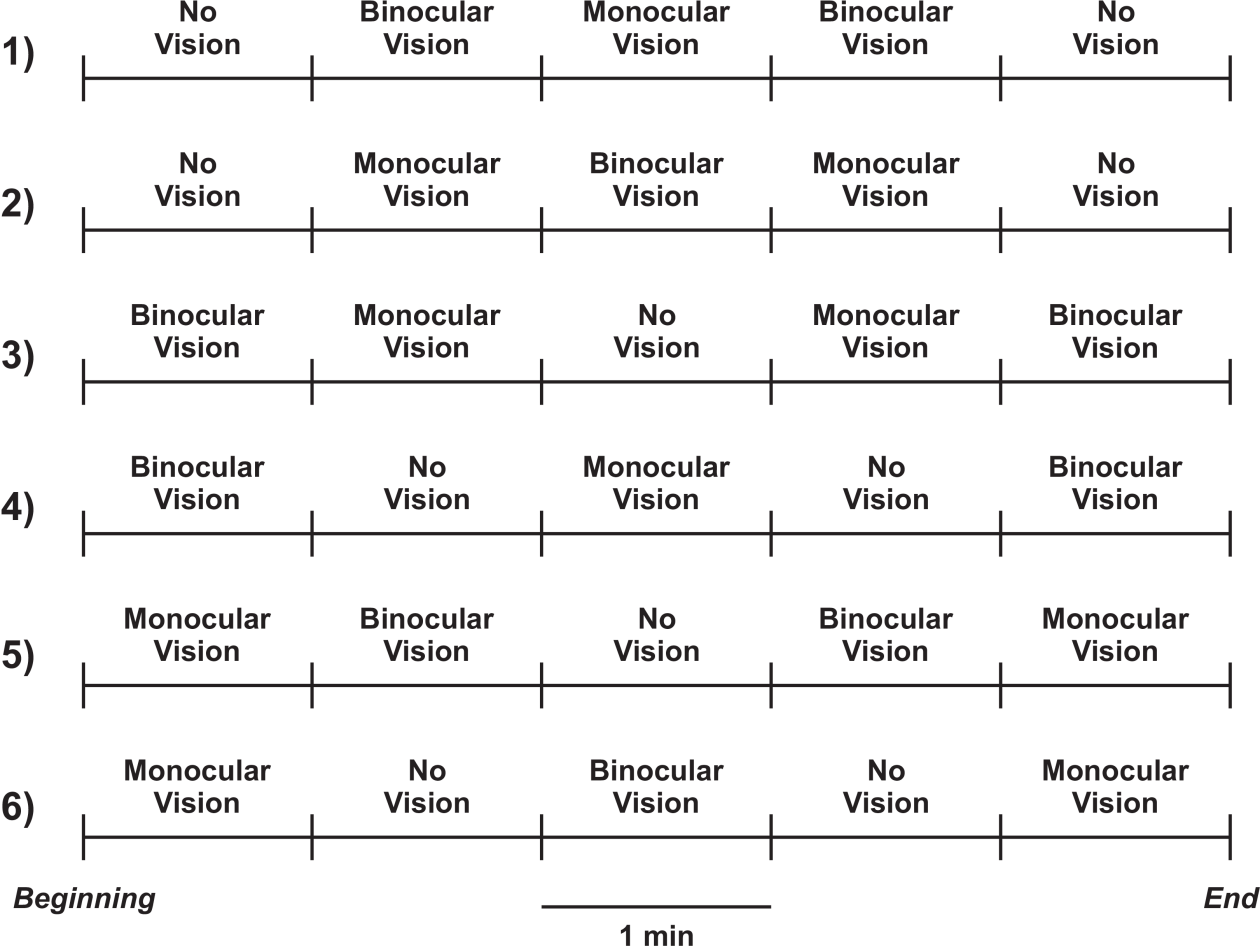
Illustration of the six visual conditions showing the lens status sequence. Each condition lasted five minutes (one minute for each lens status).

Six experimental conditions were created, as shown in Fig 1. In condition 1, for example, visual transition involved the following sequence: no vision—NV (lens opaque), binocular vision—BV (lens transparent), monocular vision—MV (lens opaque for the non-dominant eye and lens transparent for the dominant eye), binocular vision, and no vision. Each visual status lasted one minute, totaling five minutes per condition. Dominant eye was determined by a test in which participants should point to a target located 5 m away. After that they should close each eye separately and inform which eye during monocular vision provided the closest positioning of the finger to the target (i.e., dominant eye).

Participants performed one trial for each experimental condition with one-minute interval between conditions. All six experimental conditions were completely randomized within two blocks of trials. Each block of trials was performed in a different distance of the participant from the front wall (i.e., 75 e 150 cm), and they were counterbalanced among participants. Visual conditions combined with the two distances totaled twelve trials per participant. Participants were not informed about room movement and had no prior experience with the moving room.

### Data analyses

Considering that the room moved only in the AP direction, all variables were computed only in this direction. Kinematics data were filtered by a fourth-order zero lag low-pass digital Butterworth filter with a cut-off frequency of 5 Hz. Two variables were computed for each cycle of the room movement: gain and phase. For gain and phase calculation a frequency response function (FRF) at the stimulus frequency was obtained by dividing the Fourier transform of body sway by the Fourier transform of room movement. The FRF was computed for each cycle of room movement (time interval of 5 s). The FRF was averaged across visual transitions. Each visual transition repeated four times in all six experimental conditions. Then, to calculate mean values to BV to NV transition, for instance, values of this visual transition from conditions 1, 4, 5, and 6 were used (Fig 1). These mean values were used for the statistical analysis.

Gain was calculated as the absolute value of the mean FRF. A gain equals to 1.0 indicates that the body sways with the same magnitude of the moving room. Gain values above 1.0 indicate that body sway is larger than room movement and the opposite for values below 1.0. Phase was computed as the argument of the FRF and indicates the temporal relationship between body sway and room movement. Phase was calculated in radians and converted to degrees. Positive (negative) values for phase indicate that body sways ahead (behind) of moving room movement. Gain and phase data are available with this publication (S1 Supporting Information).

Because phase values seemed to vary substantially in the NV condition, we computed the standard deviation of the phase (i.e., phase variability), using phase values for the interval between the second and the tenth cycle of the room movement for each visual condition. The reason for choosing this interval was to prevent any visual transient effect due to previous visual condition.

### Statistical analyses

The first group of statistical analyses involved gain and phase as dependent variables. Based upon our experimental design, we grouped the data according to the combination of visual transitions: 1) BV to NV and NV to BV; 2) BV to MV and MV to BV; and 3) MV to NV and NV to MV. For each combination of visual transition, we run three-way repeated measures MANOVA with distance (75 and 150 cm), cycle (before-2 [B-2], before-1 [B-1], before 0 [B0], transition [T], after+1 [A+1], and after+2 [A+2]), and visual transition (1, 2, or 3) as factors. For these statistical analyses, three cycles before visual transition (B-2, B-1, and B0), the cycle correspondent to the visual transition (T) and two cycles after visual transition (A+1 and A+2) were used. We analyzed the main effects (distance, cycle, and visual transition) and the interaction between and among factors (distance*cycle, distance*visual transition, cycle*visual transition, and distance*cycle*visual transition).

For the second group of statistical analysis, the tested variable was the standard deviation of the phase (phase variability). For each combination of visual transition, we carried out three-way (2x2x2) repeated measures ANOVA with visual transition (1, 2, or 3), distance (75 and 150 cm), and interval (before and after the change in visual condition) as factors. We tested the main effects (visual transition, distance, and interval) and the interaction between and among factors (visual transition*distance, visual transition*interval, distance*interval, visual transition*distance*interval). For all comparisons, alpha value was set at 0.05.

## Results

**Fig 2:**
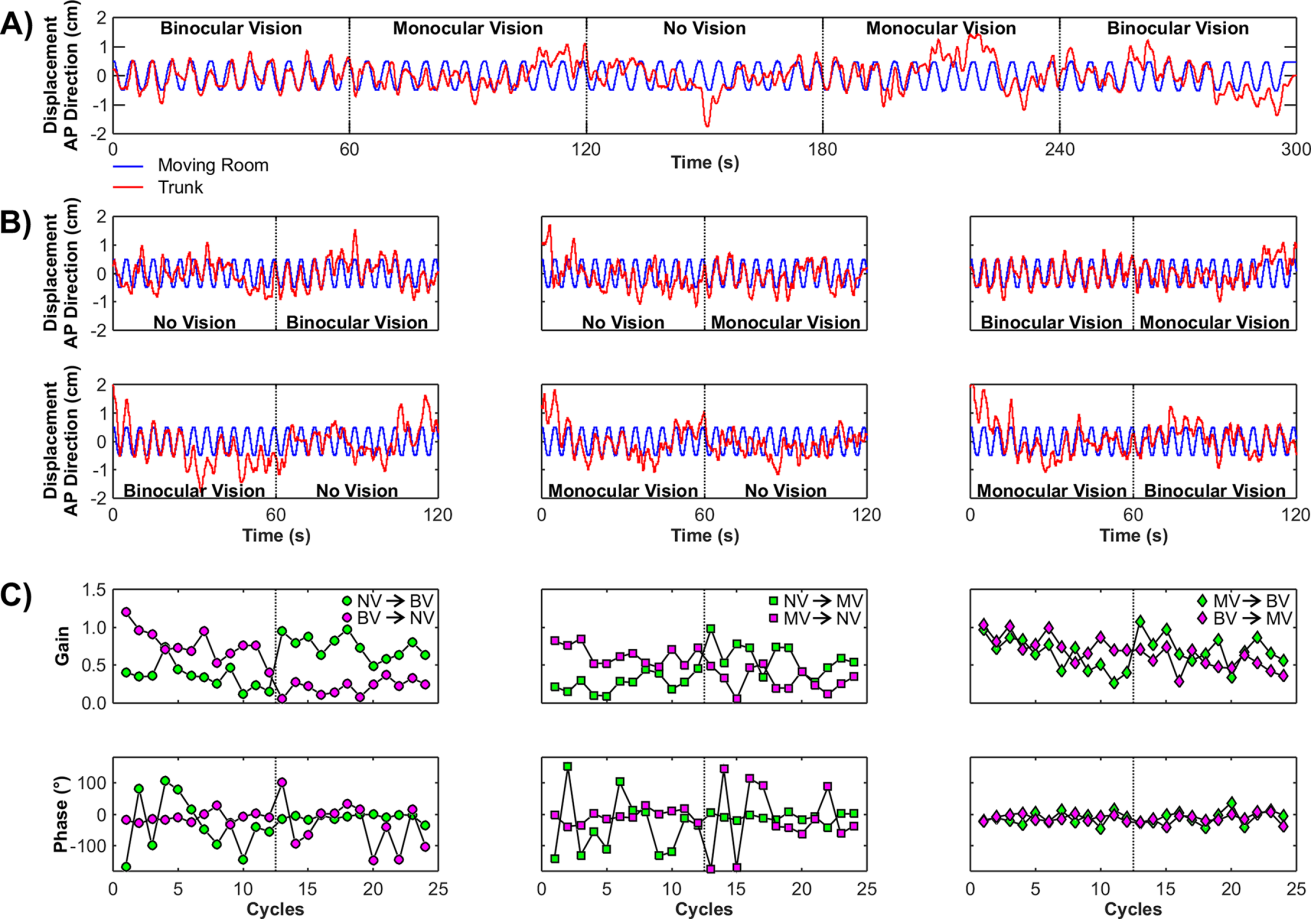
Example of time series for one participant for all visual transitions. A) Time-series for one participant showing room movement (blue line) and body sway (red line) in condition 3. B) Time-series of part of the whole trial duration (i.e., 120 s) illustrating all of the visual transitions investigated in the present study. This data is for the same participant of part A. C) Cycle-by-cycle gain (top) and phase (bottom) values for one participant (same as in parts A and B) in the different combination of visual transitions. Vertical dashed lines indicate visual transitions. [BV: binocular vision | NV: no vision | MV: monocular vision].

Visual manipulation induced correspondent postural sway in all participants. Fig 2 depicts room movement and body sway time-series for a participant throughout all experimental conditions and cycle-by-cycle gain and phase values for the different combinations of visual transitions. For each visual condition, there are twelve cycles of the moving room and it is interesting to note that phase values are quite variable for the no vision intervals.

### Binocular vision and no vision transitions

**Fig 3:**
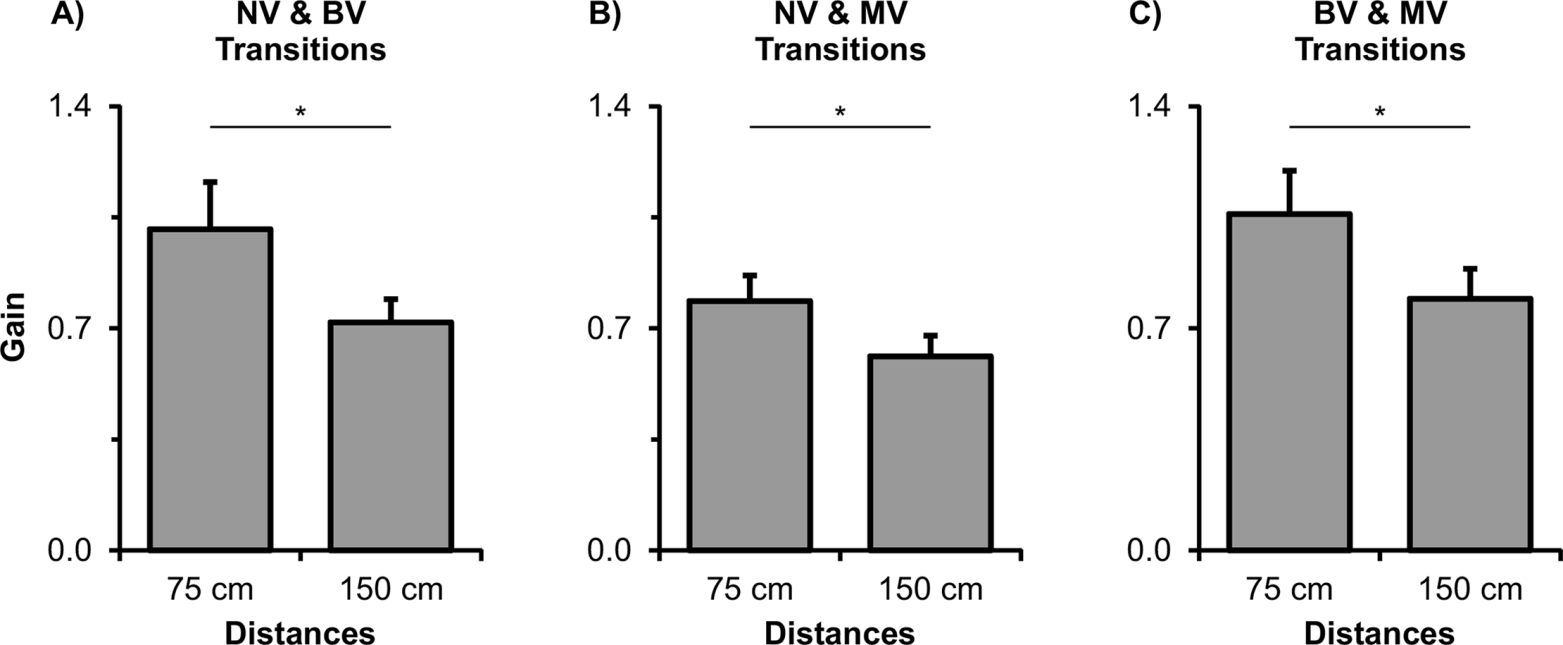
Gain mean and standard error values for both distances in the different combination of visual transitions. A) Binocular and no vision transitions; B) Monocular and no vision conditions; C) Binocular and monocular vision transitions. For mean and standard error calculation, values were collapsed from all cycles. Horizontal lines indicates pairwise differences (* p≤0.05) [BV: binocular vision | NV: no vision | MV: monocular vision].

**Fig 4:**
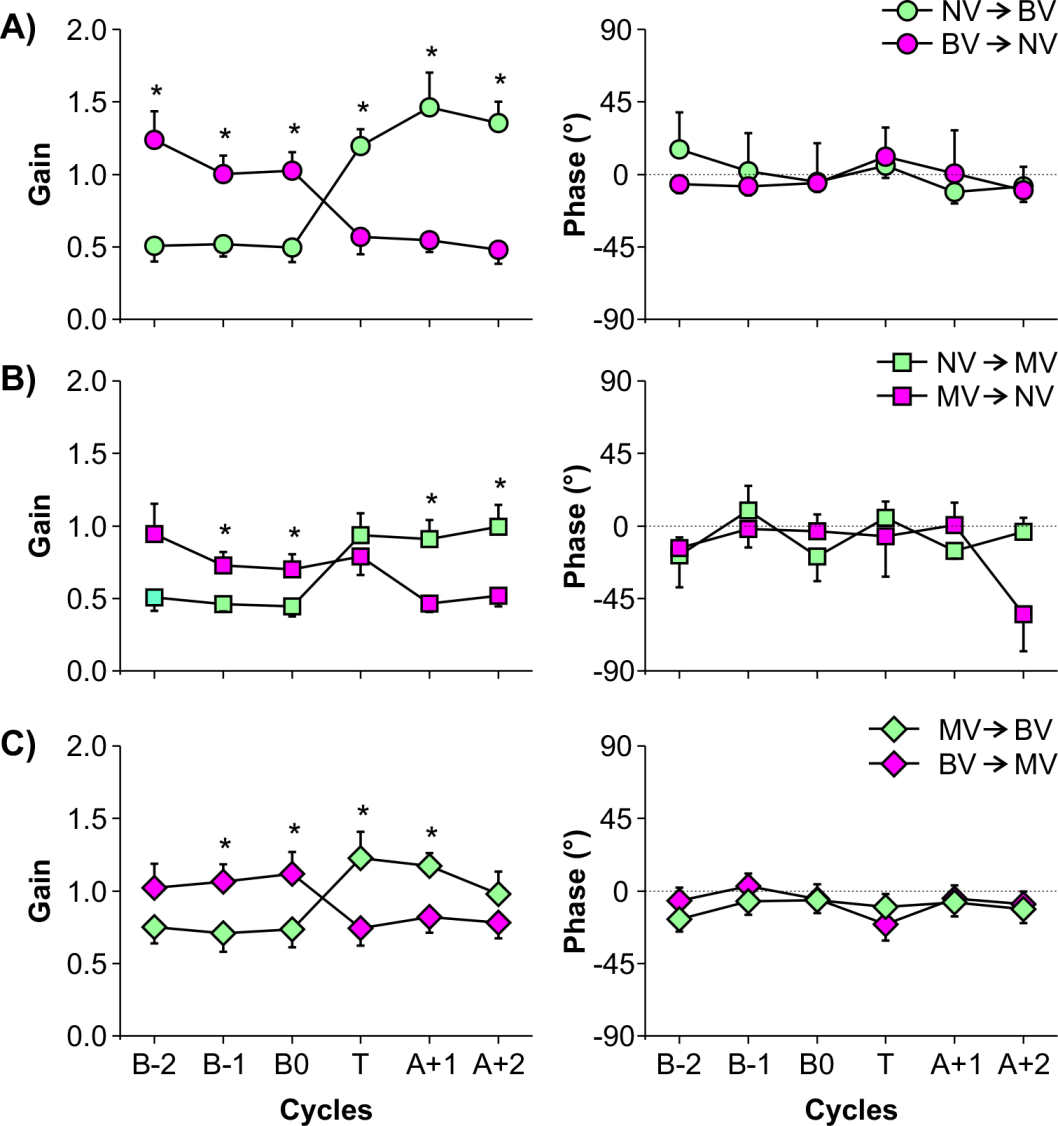
Cycle-by-cycle gain (left column) and phase (right column) mean and standard error values for the combined visual transitions. A) Binocular and no vision transitions. B) Monocular and no vision transitions. C) Binocular and monocular vision transitions. Asterisks indicate pairwise differences between visual conditions (*p≤0.05) [BV: binocular vision | NV: no vision | MV: monocular vision].

MANOVA revealed main effect of visual transition (Wilks’ Lambda = 0.237, F_2,9_ = 14.455, p = 0.002) and distance (Wilks’ Lambda = 0.481, F_2,9_ = 4.856, p = 0.037), and interaction between visual transition and cycle (Wilks’ Lambda = 0.264, F_10,98_ = 9.259, p≤0.0001). Follow-up univariate analyses revealed main effects and interaction only for gain (visual transition: F_1,10_ = 31.929, p≤0.0001; distance: F_1,10_ = 9.238, p = 0.012; and interaction: F_5,50_ = 27.213, p≤0.0001). Gain values were higher for the 75 than for the 150 cm distance (Fig 3A). Gain values were higher for the NV to BV condition (0.92 ±0.11) than for the BV to NV condition (0.81 ±0.11). It is important to note, however, that these mean gain values were obtained combining data from all cycles (NV and BV). As suggested in Fig 4A, this difference may be due to an increase in gain values for the binocular condition when it was preceded by the NV condition. ANOVA confirmed this observation by identifying a difference between gain values for BV when it was preceded by NV (F_1,10_ = 34.096, p≤0.0001). When preceded by NV, gain values were larger (1.34 ±0.15) than when it was not preceded by NV condition (1.09 ±0.14). Fig 4A shows the interaction effect between visual transitions and cycles. As can be seen, gain values reduced from BV to NV and increased from NV to BV. Most interestingly, the change in gain values was abrupt and observed right in the first cycle after the visual change.

### Monocular vision and no vision transitions

MANOVA revealed main effect of distance (Wilks’ Lambda = 0.127, F_2,9_ = 31.013, p≤0.0001) and cycle (Wilks’ Lambda = 0.608, F_10,98_ = 2.764, p = 0.005), and visual transition and cycle interaction (Wilks’ Lambda = 0.514, F_10,98_ = 3.868, p≤0.0001). Follow-up univariate analyses identified main effects and interaction only for gain (distance: F_1,10_ = 43.443, p≤0.0001; cycle: F_5,50_ = 3.819, p = 0.005; interaction: F_5,50_ = 6.209, p≤0.0001). Gain was higher for the 75 than for the 150 cm distance (Fig 3B). Gain was also higher for the transition cycle (0.86 ±0.08) than for B-1 cycle (0.60 ±0.06). Fig 4B shows the interaction effect between visual transitions and cycles. As can be seen, gain values reduced from MV to NV and increased from NV to MV. For the NV to MV transition, the change in gain values was abrupt and occurred in the first cycle after visual change. Differently, the change in gain values for the MV to NV transition occurred only in the second cycle (A+1 cycle) after visual change (transition cycle: p = 0.538).

### Binocular vision and monocular vision transition

MANOVA revealed main effect of distance (Wilks’ Lambda = 0.248, F_2,9_ = 13.666, p = 0.002) and visual transition and cycle interaction (Wilks’ Lambda = 0.390, F_10,98_ = 5.901, p≤0.0001). Follow-up univariate analyses revealed main and interaction effects only for gain (distance: F_1,10_ = 27.727, p≤0.0001; interaction: F_5,50_ = 12.464, p≤0.0001). Gain values were higher for the 75 than for the 150 cm distance (Fig 3C). For the visual transition and cycle interaction, gain values decreased from BV to MV and increased from MV to BV (Fig 3C). Again, change in gain values was abrupt and occurred in the first cycle of visual change.

### Phase variability

**Fig 5:**
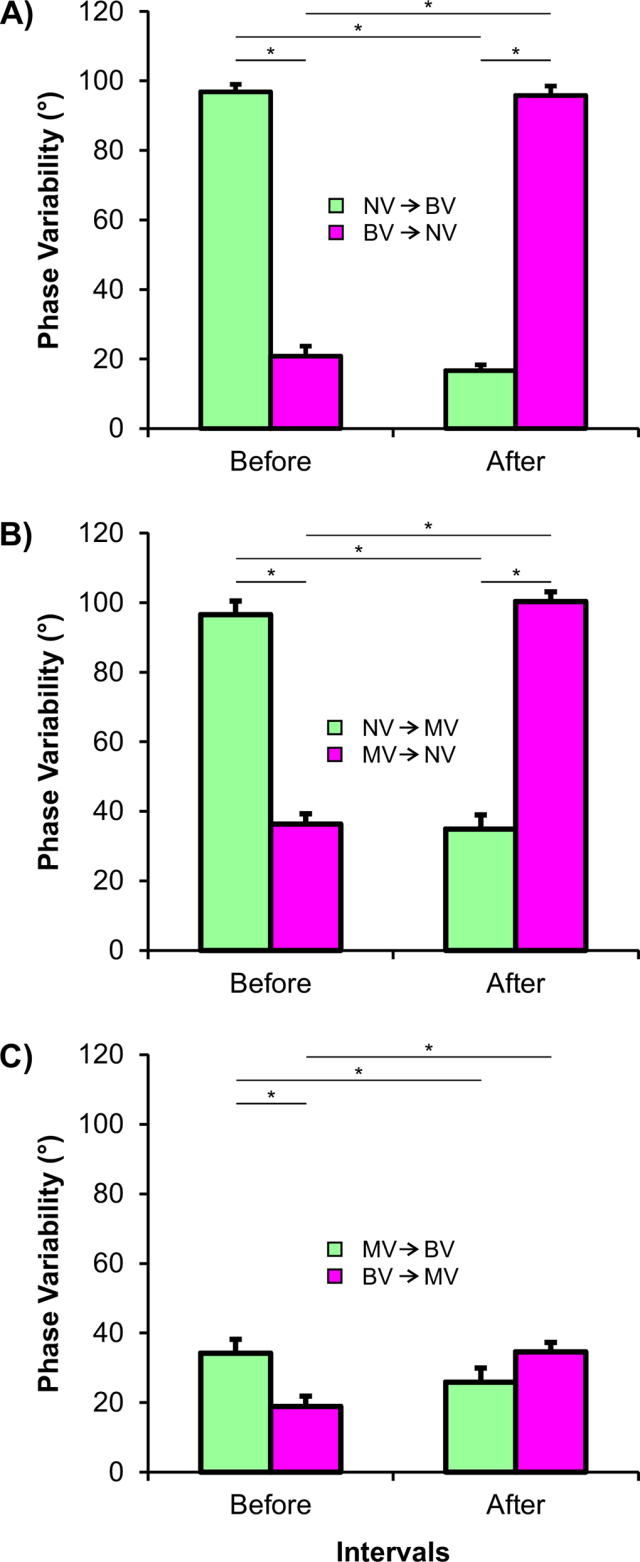
Mean and standard error for phase variability in the different visual transitions. A) Binocular and no vision transitions. B) Monocular and no vision transitions. C) Binocular and monocular vision transitions. Horizontal lines indicates pairwise differences (* p≤0.05) [BV: binocular vision | NV: no vision | MV: monocular vision].

ANOVA for the visual transition from BV and NV identified only a visual transition and interval interaction (F_1,10_ = 1882.605, p≤0.0001). Phase variability increased for the intervals without visual information (Fig 5A). The same statistical result was observed for the ANOVA involving MV and NV (F_1,10_ = 259.193, p≤0.0001). Again, phase variability increased for the intervals without visual information (Fig 5B). Interestingly, this statistical result was also observed for the ANOVA encompassing BV and MV (F_1,10_ = 17.822, p = 0.002), with phase variability increasing for the MV intervals (Fig 5C).

## Discussion

The aim of this study was to examine the effect of visual transitions in the coupling between visual information and body sway in two different distances from the front wall of a moving room. Our results indicated that the use of monocular vision and being far from the front wall of the room reduced the effect of visual manipulation on postural sway (i.e., action-perception coupling). Regarding the main question of the present study, changing visual condition resulted in an immediate change in the coupling between visual information and body sway. However, the effect of this transition was visual condition dependent, with a strong effect when transitions involved binocular and less strong effect when transitions involved monocular vision. Finally, distance from the front wall of the moving room did not interact with visual transition and it was not possible to confirm the hypothesis that adaptation would be lower and slower for the farthest distance.

Sensory reweighting has been examined by changing characteristics of a sensory modality (e.g., increasing or decreasing the amplitude and/or velocity of the visual surrounding) and looking at the postural adjustments triggered by this change in a trial-by-trial basis [5,20,21,22]. The innovative aspect of the present study lies in the fact that instead of changing the characteristics of the visual environment, the manipulation employed was subtle, changing the quality of the visual cues available during an ongoing trial in order to examine the immediate effect of visual transition on the coupling between visual information and body sway. Similarly to results from the previous studies in which stimuli amplitude and/or velocity varied, our results also showed visual reweighting due to cues coming in a monocular or binocular organization. Actually, indirect changes in postural sway responses due to monocular and binocular cues had already been observed [11], since such changes in visual cues were observed in trial-by-trial basis. Therefore, the results of the present study advance our knowledge showing an immediate effect (online reweighting) during the transition from monocular to binocular and from binocular to monocular on the coupling between visual information and body sway.

Besides showing an immediate sensory reweighting, our results also show a clear difference of the visual cues quality in the reweighting process. The online adjustment of the relative contribution of visual cues on the coupling between visual information and body sway is more effective when binocular cues are available compared to monocular ones. The transition from no vision to binocular vision condition resulted in a clear change in gain values. This indicates that the first exposure to the movement of the moving room, when binocular cues are available, leads to a quick and strong influence of vision on body sway. The opposite is also true, since the first cycle of movements of the room after removal of vision results in a sharp decrease in gain, indicating a reduction in the coupling between visual information and body sway. Also, for the transition involving monocular and binocular vision, there is a very marked change in the first cycle of visual transition.

Similarly to the binocular condition, the first exposure to the movement of the room using monocular vision also resulted in an abrupt increase in gain when preceded by no vision. However, the transition involving monocular to no vision (MV to NV) showed no such abrupt adjustment in the coupling between visual information and body sway, since gain values did not decreased as much as for the binocular condition. Rather, the expected decrease in gain occurred only in the second cycle of the moving room. Another piece of evidence regarding the asymmetry in sensory reweighting due to the binocular and monocular cues comes from the phase values. Phase values were more variable in the monocular than in the binocular vision condition. This large variability indicates a less consistent temporal relationship between room movement and body sway when visual cues are impaired reflecting a down weighting of visual information.

The delayed reduction in gain, observed in the no vision condition preceded by monocular cues, is surprising and unexpected, since there were no vision cues available and the coupling between visual information and body sway should be immediately compromised. This finding could be due to body resonance that would occur and still persevere at the same frequency of the visual stimulation even after visual cues were no longer available. However, such possible resonance effect seems not to happen when binocular vision is involved. In this case, binocular visual cues are more informative than monocular cues and when they are no longer available, sensory down weighting occurs both intense and quickly. A resonant behavior was also observed when proprioceptive information was reduced. In the absence of visual information, sway-referencing of the support surface generated a transient ~1 Hz resonating body sway when the support surface returned to and stayed at the horizontal orientation [23].

Temporal asymmetry in sensory reweighting has been observed when stimulus characteristics have been manipulated [2,3] and our results suggest that sensory asymmetry also occurs due to the quality of the sensory cues. When the sensory cue is informative but it is no longer available, its influence on postural control is abruptly ceased. Alternatively, if the cue is not sufficiently informative, the postural control system takes longer to down weight its influence, such in the case of monocular cues, and the condition of the resonance fades out only within one cycle. Such strategy is quite clever, as previously suggested [2,3], because prevent the system to change any sensory influence before making sure that such change is the optimal solution.

Our study, however, is not without limitations. Future studies should focus on specific visual switches within one trial instead of combining several visual transitions as we did in the present study. It would be interesting to have longer intervals of different visual cues to better characterize the adaptation process over a long time scale, since it might present slow and fast adaptive process of sensory reweighting [2,9]. Longer trials also could uncover issues such as the reverberation contribution suggested to happen in our results.

Finally, it is important to consider the relevance of sensory reweighting in clinical research and its application. As shown in our study, the quality of visual cues played an important role in determining the adaptive characteristics of sensory weighting. In this case, it would be interesting to investigate how such a process is established in patients with sensory deficits. For instance, patients with vestibular deficits are known for their inability to compensate for inconsistent visual and proprioceptive information [24]. Considering that under monocular vision, healthy individuals increase the contribution of the vestibular system to postural control [10], patients with vestibular deficits might rather increase visual gain for monocular cues and may show a faster reweighting process when monocular cues are involved in the visual transitions.

In summary, we conclude that the paradigm of manipulating the quality of the visual cues by changing the amount of visual information available allowed identifying the process of adjusting the relative contribution of the visual information to postural control in young adults. The findings indicate that sensory reweighting is observed immediately after the visual transition and it is more effective when involving binocular cues as compared to monocular ones, suggesting that in the presence of binocular vision, the weight of the visual information is much stronger than in the presence of monocular vision. However, this immediate sensory reweighting in both binocular and monocular is affected similarly by the distance between the individual and the front wall of the room. Independently of the amount of visual information available, the effect of visual manipulation on postural sway and sensory reweighting is stronger when individuals are closer to the front wall of the room.

## Supporting Information

S1 Supporting Information Spreadsheet named "Gain & Phase" contains means and standard errors from the statistical analyses involving visual transitions. Spreadsheet named "Phase Variability" contains means and standard errors from the statistical analyses involving phase variability.
(XLSX)

## References

[1] Barela JA, Weigelt M, Polastri PF, Godoi D, Aguiar SA, Jeka JJ. Explicit and implicit knowledge of environment states induce adaptation in postural control. Neurosci Lett. 2014;566: 6–10. 10.1016/j.neulet.2014.02.029 24582899

[2] Jeka JJ, Oie KS, Kiemel T. Asymmetric adaptation with functional advantage in human sensorimotor control. Exp Brain Res. 2008;191: 453–463. 10.1007/s00221-008-1539-x 18719898 PMC2731555

[3] Oie KS, Kiemel T, Barela JA, Jeka JJ. The dynamics of sensory reweighting: a temporal asymmetry. Gait Posture. 2005;21: S29.

[4] Oie KS, Kiemel T, Jeka JJ. Multisensory fusion: simultaneous re-weighting of vision and touch for the control of human posture. Cogn Brain Res. 2002;14: 164–176. 10.1016/s0926-6410(02)00071-x 12063140

[5] Carver S, Kiemel T, Jeka JJ. Modeling the dynamics of sensory reweighting. Biol Cybern. 2006;95: 123–134. 16639582 10.1007/s00422-006-0069-5

[6] Horak FB, Macpherson JM. Postural equilibrium and orientation In: Rowell LB, Shepherd JT, editors. Handbook of Physiology: a critical, comprehensive presentation of physiological knowledge and concepts. New York: Oxford University Press; 1996 pp. 255–292.

[7] Paulus WM, Straube A, Brandt T. Visual stabilization of posture: physiological stimulus characteristics and clinical aspects. Brain. 1984;107: 1143–1163. 6509312 10.1093/brain/107.4.1143

[8] Romberg MH. A manual of the nervous diseases of man. London: The Sydenham Society; 1853.

[9] Polastri PF, Barela JA, Kiemel T, Jeka JJ. Dynamics of inter-modality re-weighting during human postural control. Exp Brain Res. 2012;223: 99–108. 10.1007/s00221-012-3244-z 22965550

[10] Jessop D, McFadyen BJ. The regulation of vestibular afferent information during monocular vision while standing. Neurosci Lett. 2008;441: 253–256. 10.1016/j.neulet.2008.06.043 18582533

[11] Moraes R, Lopes AG, Barela JA. Monocular vision and increased distance reducing the effects of visual manipulation on body sway. Neurosci Lett. 2009;460: 209–213. 10.1016/j.neulet.2009.05.078 19501130

[12] Barela JA, Sanches M, Lopes AG, Razuk M, Moraes R. Use of monocular and binocular visual cues for postural control in children. J Vis. 2011;11: 10. 10.1167/11.12.10 22004694

[13] Freitas PB Júnior, Barela JA. Postural control as a function of self- and object-motion perception. Neurosci Lett. 2004;369: 64–68. 15380309 10.1016/j.neulet.2004.07.075

[14] Barela AM, Barela JA, Rinaldi NM, Toledo DR. Influence of imposed optic flow characteristics and intention on postural responses. Motor Control. 2009;13: 119–129. 19454775 10.1123/mcj.13.2.119

[15] Guerraz M, Thilo KV, Bronstein AM, Gresty MA. Influence of action and expectation on visual control of posture. Cogn Brain Res. 2001;11: 259–266. 10.1016/s0926-6410(00)00080-x 11275487

[16] Aguiar SA, Gramani-Say K, Lopes AG, Barela JA. Dual task interferes with sensorimotor coupling in postural control. Psychol Neurosci. 2014;7: 593–599.

[17] Lê TT, Kapoula Z. Distance impairs postural stability only under binocular viewing. Vision Res. 2006;46: 3586–3593. 16899270 10.1016/j.visres.2006.06.018

[18] Godoi D, Barela JA. Body Sway and sensory motor coupling adaptation in children: effects of distance manipulation. Dev Psychobiol. 2008;50: 77–87. 18085560 10.1002/dev.20272

[19] Soames RW, Atha J. The spectral characteristics of postural sway behavior. Eur J Appl Physiol. 1982;49: 169–177. 10.1007/BF02334065 6981506

[20] Rinaldi NM, Polastri PF, Barela JA. Age-related changes in postural control sensory reweighting. Neurosci Lett. 2009;467: 225–229. 10.1016/j.neulet.2009.10.042 19840830

[21] Jeka JJ, Allison LK, Kiemel T. The dynamics of visual reweighting in healthy and fall-prone older adults. J Mot Behav. 2010;42: 197–208. 10.1080/00222895.2010.481693 20501430

[22] Assländer L, Peterka RJ. Sensory reweighting dynamics in human postural control. J Neurophysiol. 2014; 10.1152/jn.00669.2013 PMC4044370 24501263

[23] Peterka RJ, Loughlin PJ. Dynamic regulation of sensorimotor integration in human postural control. J Neurophysiol. 2004;91: 410–423. 13679407 10.1152/jn.00516.2003

[24] Nashner LM, Black FO, Wall C. Adaptation to altered support and visual conditions during stance: patients with vestibular deficits. J Neurosci. 1982;2: 536–544. 6978930 10.1523/JNEUROSCI.02-05-00536.1982 PMC6564270

